# Evaluation of the use of a screening tool by community and religious leaders trained by a distance learning course

**DOI:** 10.1186/1940-0640-8-S1-A16

**Published:** 2013-09-04

**Authors:** Ana Paula L Carneiro, Paulina CAV Duarte, Maria Lucia O Souza-Formigoni

**Affiliations:** 1 Federal University of São Paulo / UNIFESP, São Paulo, Brazil; 2 Brazilian National Secretary on Drug Policy/SENAD, Brasília, Brazil

## 

Considering the need of training for community and religious leaders to deal with alcohol and other drug-associated problems, a distance learning course named Faith in Prevention was developed and offered to 10,000 Brazilian religious and community leaders. We used the following indicators to assess the course’s effectiveness: participant interest in the course, performance of the proposed activities, and use and applicability of the screening and brief intervention (BI) techniques. One year after the end of the 1^st^ edition of the course and eight months after the end of its 2^nd^ edition, an online questionnaire was sent to all approved participants (7387). The instrument contains questions on the use of the techniques, the implementation problems faced after the course, and their activities to disseminate the knowledge gained to other community or religious leaders. In all editions, adherence was high; about 80% of those who started the course went on to finish it. 1815 people (24.6%) agreed to answer the online questionnaire. Most of them (68.5%) considered the course was very important to their daily activities and felt very motivated to use the screening tools (57%) and BI (53.5%). Regarding dissemination, 54% of them had already trained between one to 20 people, and 15% reported having trained between 21 to 50 people. Most of the participants had already applied the ASSIST (61%), BI for alcohol (78%) and BI for tobacco (67%). We also found some differences between the participants in the two editions as regards the importance of the techniques, their incorporation in their daily activities, and colleague support. The participants from the 2^nd^ edition were aware of the importance of SBI and used the techniques more often than those from the 1^st^ edition. Results indicate religious and community leaders have been using and disseminating the techniques learned via the Faith in Prevention distance learning course.

